# Influence of new sulfur-containing fertilizers on performance of wheat yield

**DOI:** 10.1016/j.sjbs.2021.04.073

**Published:** 2021-04-30

**Authors:** Meruyert Kurmanbayeva, Tolganai Sekerova, Zhanar Tileubayeva, Tursynbek Kaiyrbekov, Adil Kusmangazinov, Shermakhan Shapalov, Aigul Madenova, Mukhambetkali Burkitbayev, Nadezhda Bachilova

**Affiliations:** a Al-Farabi Kazakh National University, Kazakhstan; b Kazakh National Women's Teacher Training University, Kazakhstan; c M. Auezov South Kazakhstan University, Kazakhstan; d Institute of Plant Biology and Biotechnology, Kazakhstan

**Keywords:** Wheat, Sulfur-containing fertilizers, Early ripeness, Rust, Resistance, Productivity

## Abstract

Wheat is the main cereal crop in Kazakhstan and fertilizers play an important role in enhancing harvest growth. In this study, the impact of new sulfur-containing fertilizers on the growth and yield of wheat was evaluated, and the resistance of varieties to *Puccinia triticina* Erikss was also investigated. (also known as *Puccinia recondite* Rob. ex Desm.) for recommendations in agriculture. The study was conducted from 2017 to 2020 in a nursery and greenhouse. The sulfur-containing fertilizer contains nutrients that allow you to extend the duration of absorption by the plant, thereby extending the period of their availability to plants, compared to conventional preparations. By encapsulating molten elemental sulfur and impregnating with a solution of calcium polysulfide, a long-acting compound based on amorphous and monocalcium phosphate was developed. The sulfur is in a water-soluble sulfate form, which, in turn, is slowly oxidized by bacteria and retained in the soil. Three different types of the developed sulfur-containing nano-particle have been used to test in greenhouses and nurseries: powdered, pasty sulfur-containing composition, and a solution of calcium polysulfide. The results showed that the use of powdered and dissolved sulfur-containing fertilizers contributed to the early ripeness and increased productivity of wheat. Wheat varieties were tested for the presence of key Lr genes that determine resistance to brown rust. The Omskaya 29 sample showed an immune response according to phytopathological assessment, and molecular screening revealed four resistance genes. The new sulfur-containing product is recommended for improving wheat productivity in agriculture, and the Omskaya 29 variety can also be used as a valuable breeding material resistant to brown rust.

## 1 Introduction

Kazakhstan has unique natural conditions for the production of cereal crops, first and foremost, unsurpassed baking qualities of soft and hard wheat varieties. However, these opportunities are not fully realized. In most cases, the dynamic balance in the ecological system is disturbed as a result of intensive mechanical treatments: the soil, plant, atmosphere, also, the biogeochemical cycle of substances changes (Dhiman et al., 2019). One of the main and necessary conditions for obtaining high wheat yields is the use of high-quality seed material with effective compounds, on which the germination of plants depends (Erdem et al., 2016). The intensification of production involves the use of various drugs to optimize plant nutrition and pesticides to control pests, diseases, and weeds in modern agriculture. The perfection of the existing forms of the agricultural system is based on the widespread preparation application and means for the plant protection and reproduction of soil fertility, as well as the introduction of differentiated tillage systems taking into account the biological requirements of plant culture (Stankowski et al., 2019). These days, the most promising area is the use of pre-sowing seed treatment with nano-particles, as evidenced by the growth in sales volumes. A feature of the action of active substances is that they intensify the physiological and biochemical processes in plants and increase yield, the resistance to stress at the same time. Such regulators include natural and synthetic substances, which in small doses actively affect the metabolism of plants (Burkitbayev et al., 2021). Intensive technology of crop cultivation provides for the full realization of the potential capabilities of plants in the formation of high yields with good quality. The resistance of the studied varities is confirmed by the data of the ionic balance of Na+, K+ and Ca2+ in primary wheat roots (Terletskaya et al., 2019). In the practice of world agriculture, new high-yielding varieties, science-based crop rotations, rational use of mineral compositions, and plant protection products are recognized as the main factors for increasing the harvest. These techniques require large energy and material costs and they are not always environmentally safe. An acute problem of modern crop production is currently the production of environmentally friendly agricultural products and the reduction of man-made load on the biogeocenosis (Monostori et al., 2017).

Leaf rusts inflict the most distributed and devastating diseases of agricultural plants due to their adaptability notably to new resistant cultivars. *Puccinia recondita* is one of the fungi representatives able to withstand and overcome *Triticum aestivum* with many rust-resistant genes (Xu et al., 2005, Hiebert et al., 2005).

The diseases infected by *P. recondita* is the paramount obstacle of wheat to deal with. It is the reason for a massive loss of productivity in agriculture. Infected leaves are dropped before their time in consequence leading to the deceleration of all biochemical processes in plant metabolism. Humongous yield losses caused by an epidemic form of wheat rusts are the crucial biotic factors. The diseases are varied by ever-changing, adaptable rust varieties and capable to deal with approaches in making crops more resistant. Rust representatives are easily distributed and they can penetrate the plant leaf with no effort (Babar et al., 2010, Bouftass et al., 2010). Hence, agricultural plants always should be reinforced with extra compounds in addition to genetic engineering.

Yield losses caused by *P. triticina* is an important issue of agriculture. Leaf rust which is a foliar disease is derived from premature senescence and poorly developed kernels. This leads to severe damage in wheat production infecting upper leaves. The rusting hinders wheat productivity during the grain filling period leading to small kernels and early loss of rusted leaves. There is another species of fungus that can reach the epidemic level of *T. aestivum*. Commonly breeders and pathologists deploy race-specific resistance genes, however, they are short-lived in nature (McCallum et al., 2007, Kolmer and Oelke, 2006). The number of rust-resistant genetic materials should be increased even there are more than 45 known genes.

It is known that sulfur helps to slow down the oxidative processes in plants with an increase in the reduction processes, while cereal crops increase their viability and improve the quality of grain. Researches about the effect of sulfur and calcium reveal the effectiveness of using sulfur to increase productivity (Ivanitsky, Ya.V., 2011, Maslova et al., 2008, Maslova, 2008) since the lack of sulfur in grain significantly impacts the production and quality of wheat grains (Zhao et al., 1999). The nitrogen cannot be used effectively without sulfur and the protein content cannot reach its full potential in terms of yield (Sahota, 2006, Ryant and Hřivna, 2004, De Ruiter and Martin, 2001). Also, sulfur is a component of several major compounds in crops, so the lack of sulfur is a limiting factor not only for growth and seed yield but also for poor product quality (Singh, 2003). Limiting the availability of sulfur contributes to the synthesis of low protein content (Flæte et al., 2005), reduces the size and quality of wheat grains due to the cessation of the formation of disulfide bonds formed from the sulfhydryl groups of cysteine (Gyori, 2005, McGrath, 2003). Sulfur-containing wheat grain, measured as the concentration of sulfur in addition to nitrogen concentration is the key to the seed quality (Karimi and Mohsenzadeh, 2015, Geiger, 2009, Whitesides, 2005) and the lack of it leads to a reduction of productivity. The results demonstrate a similarity between CuO or ZnO in wheat plants with higher root toxicity correlated with a smaller size of sulfur nanoparticles (Hasan et al., 2018, Dimkpa et al., 2013, Tea et al., 2007).

Besides, the yield is affected by the resistance of wheat varieties to rust fungus. It has been planned to offer resistant varieties for selection against rust fungi. The reason for that is the production and use of rust-resistant varieties in agriculture is an important issue. Polymerase chain reaction protocols were optimized to identify carriers of such brown rust resistance genes as Lr68, csGS marker; Lr19/Sr25, PSY-E1 marker; Lr35/Sr39, Sr39#50 marker; Lr37/Yr17/Sr38 Ventriup/ LN marker; Lr39 xgwm 210 marker and Lr28 Wmc 313 marker.

The deterioration of the phytosanitary situation has led to a sharp decline in quality indicators and yield, which hinders the development of wheat in all growing seasons. The fungal diseases are the most common and harmful among diseases of cereal crops (Pranckietienė et al., 2019). The total damage from fungal diseases, including rust, as a result of which the soft wheat crop is estimated annually worldwide in billions of dollars. According to experts of the Food and Agriculture Organization of the United Nations (FAO), the annual global loss of food from diseases and pests of crops is up to 70 million tons (El-Salam et al., 2015). The damage by phytopathogenic fungi is a serious problem for the countries of Central Asia because agriculture is considered as one of the main factors of the economy and according to the FAO, this region is characterized by a higher level of bread consumption per capita (about 200 kg per year) as well. It cut back on production expenditures, which is an economic factor that negatively affects the sustainable development of agriculture (Maslova et al., 2008).

One of the factors that reduce the yield is the disease of brown rust (Ivanitsky, Ya.V., 2011, Sahota, 2006, Zhao et al., 1999). In the shortest possible time, when the climatic conditions of the environment are favorable, this pathogen intensively spreads and affects wheat and other crops of *Triticum, Aegilops, Elymus, Agropyron*, etc. (Ryant and Hřivna, 2004, De Ruiter and Martin, 2001).

The most harmful brown rust of wheat (*Puccinia recondita* f. sp. tritici Erikss), widespread in all arable lands in the world, reduces the yield of grain crops by 5–10% annually, and epiphytosis in these years leads to a loss of 50–70%. According to scientists, this type of rust causes economic losses at higher costs than yellow and stem rust (Flæte et al., 2005, Singh, 2003).

Mass development of brown rust of wheat is found in all arable lands of Kazakhstan. The identification of lines of soft and durum wheat tested from the control nursery for the presence of key Lr genes that determine resistance to brown rust was carried out. Regard to this study aimed to identify lines of soft and durum wheat tested from a controlled nursery for the presence of key Lr genes that determine resistance to brown rust. Also, most of the chemicals used in modern agricultural production are artificial and are not destroyed either by the enzymatic systems of plants or by physical or chemical approaches. This leads to accumulation in the harvested crop, consequently, in the body of people and animals.

The solution to this problem can be the use of a multifunctional long-acting nano-sulfur, which contains nutritional elements that access to extend the duration of the absorption by the plant, thereby extending the period of their availability to plants, compared to conventional fertilizers. Therefore, the purpose of this study is to evaluate the effectiveness of new sulfur-containing agrochemicals on the growth, yield, and resistance of wheat to brown rust, in various growing conditions.

## 2 Materials and methods

### 2.1 The synthesis of calcium polysulfide for the treatment of plants

A solution of calcium polysulfide synthesized by chemists from the faculty of the chemistry of al-Farabi Kazakh National University on local raw materials in a pilot reactor with a volume of 150 L has been used for processing. The raw materials are petroleum sulfur extracted during the oil refining from the Tengiz field (Western Kazakhstan) and calcium oxide obtained by firing limestone-shell rock from the Beineu field (Western Kazakhstan). A solution of calcium polysulfide with a density of 1.24 g/cm^3^ was obtained. A patent of the Republic of Kazakhstan No. 4817 dated 20.07.2020 was received for the method of obtaining calcium polysulfide.

The figures show diffractograms of the initial sulfur, calcium oxide from limestone-shell rock, and the finished product. X-ray phase analysis was carried out on a modernized DRON-3 M diffractometer using CuKα-radiation with software for radiographs obtained in the shooting range of 20 (angles) from 10 to 70^0^, step-0.05, speed-2 g/min, the maximum intensity of 3000 imp/sec. The interpretation of the diffractograms was carried out using data from the ICDD card file: PDF2 powder diffraction database (Powder Diffraction File) and diffractograms of impurity-free minerals. The content was calculated for the main phases. Conditions for shooting diffractograms: U = 35 kV; I = 20 mA; shooting θ-2θ; detector 2 deg/min.

**Fig. 1:**
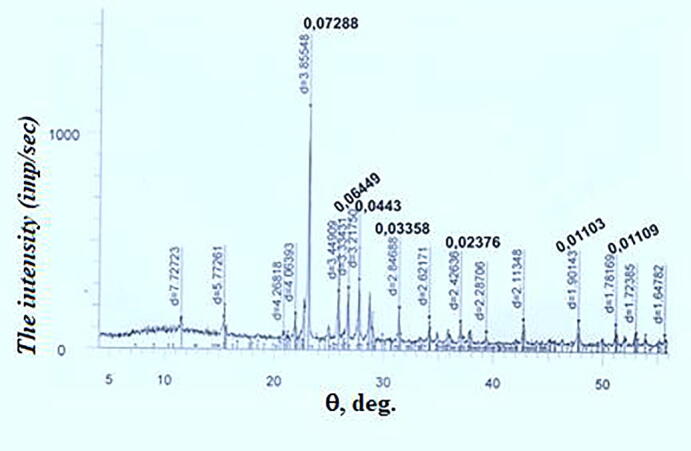
Radiograph of petroleum sulfur.

The diffractogram illustrates the sulfur of the Tengiz deposit is represented by two polymorphic modifications: S_8_ and S_13_ (Fig. 1).

### 2.2 Production of calcium oxide from limestone-shell rock

**Fig. 2:**
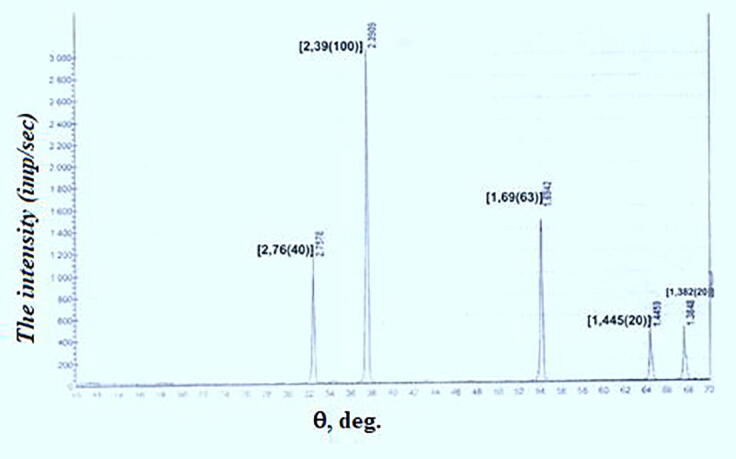
X-ray image of quicklime (CaO) obtained by firing limestone-shell rock of the Beineu deposit.

Fig. 2 shows a diffractogram of calcium oxide obtained by firing the shell-limestone at a temperature of 950 °C in a muffle furnace. The X-ray shows all the CaO lines: d/n = 2.7578; 2.3909; 1.4459 and 1.3848 Å. Among them, the analytical line with d/n = 2.3909 Å – 3000 imp/sec has the highest intensity. A comparison of its value with the reference CaO (its indicators are placed in square brackets on the X-ray image) indicates the crystallization of CaO from the shell almost in pure form without a solid solution; the density is 3.33 g / cm^3^.

Thus, the calcium oxide obtained from the shell corresponds to the reference sample of calcium oxide and it does not contain impurities, which ensures the production of pure calcium polysulfide from local Kazakhstan raw materials.

### 2.3 Synthesis of calcium polysulfide

**Fig. 3:**
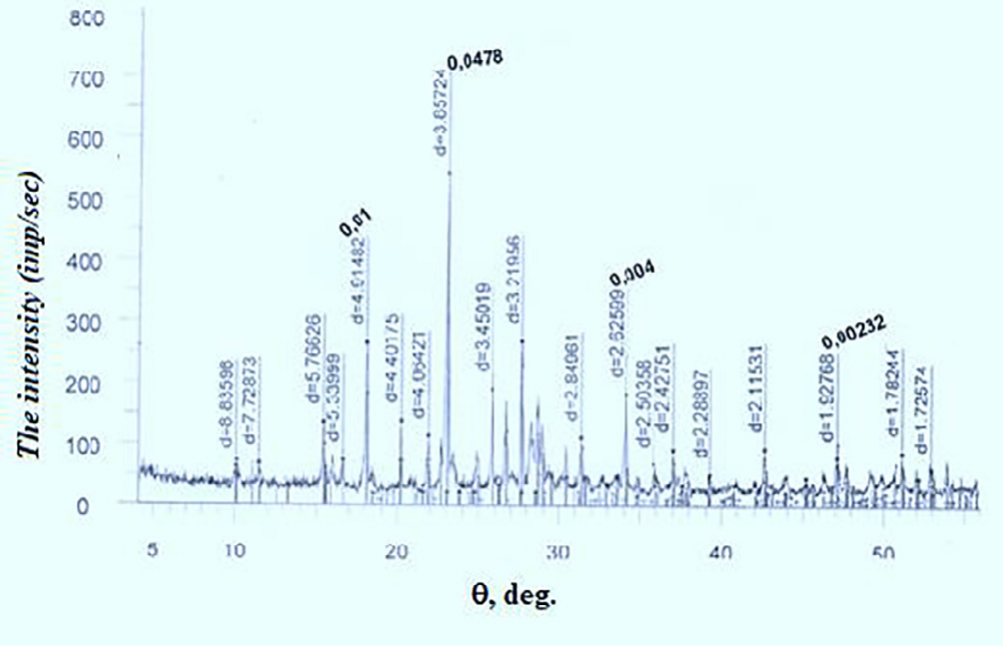
Radiograph of the decomposition product of calcium polysulfide.

A freshly prepared aqueous solution of calcium polysulfide of 2% concentration was used for the treatment of plants. Fig. 3 shows a diffractogram of the decomposition products of calcium polysulfide in water, which, in addition to the diffraction maximum belonging to elemental sulfur, contains a maximum of calcium hydroxide, which is a product of the decomposition of calcium polysulfide in water as well.

Thus, the aqueous solution of the compound contains elemental sulfur and calcium hydroxide. The content of elemental sulfur in calcium polysulfide with a density of 1.24 g/cm^3^ is 160–180 g/l, and the content of sulfur in a 2% solution is 3.2 – 3.6 g/l. The ratio of sulfur to calcium hydroxide in the solution is 1.47–1.49 and it is constant.

At the first stage of decomposition of calcium polysulfide diluted with water, primary nanoparticles with an average size of 20 nm are formed, which are stable for a certain (10–15 min) time, then the nanoparticles begin to assemble into larger particles (aggregates) with sizes ranging from 80 nm to 400 nm. Based on this, the treatment of plants was carried out immediately after diluting the concentrated solution of calcium polysulfide with water, which ensured the preservation of the nanodisperse state of sulfur and calcium hydroxide particles, which are convenient for assimilation by plants.

A further increase in the time leads to the fact that aggregates with sizes from 80 nm to 400 nm gradually (within 6–7 h) are enlarged and form agglomerates in the range from 30 µm to 100 µm, which settle out of the solution, making it unsuitable for processing plants.

The presence of nano dispersed particles of calcium hydroxide in the 2% solution, which is carbonized in the nano dispersed state on the surface of plants, in a mixture with nano sulfur significantly enhances plant growth.

Thus, during processing, two types of nanoparticles are deposited – sulfur with a size of 20–40 nm and calcium carbonate particles with a size of 25–50 nm, which makes it possible to classify the drug as a growth stimulator.

### 2.4 The experiment in greenhouse conditions

The research was conducted in 2017–2020 in the conditions of an innovative greenhouse at the al-Farabi Kazakh National University. The object is wheat (*Triticum* L.), and three different types of sulfur-containing nano-particle have been used as fertilizers during the experiment: 1-powdered sulfur-containing composition, 2-pasty sulfur-containing chemicals, 3-A solution of calcium polysulfide. The 2% solutions were used to study the effect of a sulfur-containing product on seed germination. In the control group, water has been applied. Pretreatment of seeds was carried out in sealed glass vials, where 100 dry grains of the same size were placed, which were immersed in 1 L of solution. The seed holding time was 15 min. Then the seeds were planted. At the end of the time, the percentage of sprouted seeds was calculated. Wheat seeds were treated with a solution of 1.24 g/cm^3^ is 160–180 g/l, and the sulfur content in the 2% solution is 3.2–3.6 g/l. The studies were carried out in 3-fold repetition. All measurements were carried out with 3-fold biological repetition. Root treatment was performed 3 times every 10 days. During the phenological observation, the number of seedlings and the height of the plants were taken into account. From the beginning of the formation of the wheat growth phase, the morphological structures of wheat were examined until full ripeness. For morphological descriptions was measured by the length of coleoptile, first leaf, all internodes, and length of flag leaf, spike, do a thorough structural analysis and determine the productivity of wheat. The study was conducted according to the generally accepted method. A structural analysis was carried out in four variants to assess the impact of new sulfur-containing agrochemicals on the growth, yield, and resistance of wheat to brown rust. Statistical analysis of the obtained data was processed in the R-Studio software.

### 2.5 The experiment in the nursery

Genetic studies of samples of wheat varieties were grown in the nurseries of the Scientific Research Institute of Plant Biology and Biotechnology. To test the resulting nano-sulfur, the nurseries used wheat lines treated with new compounds and examined for the presence of key Lr genes that determine resistance to brown rust. For planting wheat, the same size of wheat grains was selected, for the quality and reliability of the experiment. Daily pheno-monitoring was carried out after planting wheat grains in various variants. The experiment was carried out with a 3-fold repetition.

The objects of research are 10 samples of winter and spring wheat. Phytopathological assessment of rust resistance of the experimental wheat material was carried out according to the method McIntosh et al. (1995). According to this method, the percentage of infection spread and the infectious type of disease were determined (0 – immune, R – resistant, MR – moderately resistant, MS – moderately susceptible, S – susceptible). The Morocco wheat variety is used as a universally susceptible standard.

When screening for brown rust resistance, 2 indicators were evaluated: the type of reaction and the degree of leaf damage. The Stakman et al. (1962) scale was used in experiments to assess seedling resistance to brown rust in a greenhouse, according to which, for the type of infection «0», the reaction is immunity with no visible symptoms; «;» – high resistance, hypersensitivity reaction; 1 - resistance, small pustules with necrosis; 2 – medium resistance, small and medium pustules without chlorosis; 3 – medium sensitivity, medium-sized pustules without chlorosis; 4 – sensitivity, large pustules without chlorosis. A mixture of mushroom spores collected from susceptible Kazakh wheat varieties was used as an inoculum.

Infection of plants was carried out in the wheat tillering phase with a suspension of uredospores of the fungus with the addition of one drop of Twin-80 (surfactant) for better adhesion of spores to wheat leaves. After infection, a wet chamber was created on the crops by covering the plants with plastic wrap for 16 h. After 12 days, wheat rust resistance was evaluated 3 times with a frequency of 7–10 days.

In laboratory conditions, studies on resistance to brown leaf rust were carried out using a benzimidazole reagent and light filters according to the method of Mikhailova and Kvitko (1979). The Mains and Jackson (1926) scale was used to determining the type of immunity to the disease. The reaction to infection of leaf segments with brown rust was taken into account on day 8 in points, where:

0 – no symptoms of the lesion-immune sample; 0 – 1-E. p.- single pustules – «hypersensitivity» reaction with the manifestation of necrosis-relatively immune; 1-very small pustules, surrounded by necrosis-highly resistant; 2-medium-sized pustules, surrounded by necrosis and chlorosis-moderately stable; 3-medium-sized pustules without necrosis-moderately susceptible; 4-large pustules, often merging, no necrosis-highly susceptible; X-pustules of different sizes-heterogeneous type of lesion-heterogeneous; When assessing the disease, the following gradation is used: the reaction with points from 0 to 1 is considered stable, 2 is an intermediate reaction; 3, 4 is a susceptible reaction.

Genomic DNA extraction from plant material was performed from wheat seedlings based on the CTAB method of genomic DNA extraction, using 5-day seedlings according to the method (Riede and Anderson, 1996). The polymerase chain reaction (PCR) method according to the Chen (1998) Protocol is used to identify carriers of resistance genes. Amplification is carried out on the thermocycler Bio-Rad T100 Thermal cycler (USA), a program of amplification was chosen depending on the identified Lr resistance gene. µl of 10x buffer for Taq polymerase, µl of dNTP (1 mm of each nucleotide), 0.2 µl of each primer, 0.2 µl of Taq polymerase, 6.4 µl of MQ-H20. To separate fragments of amplified DNA, electrophoresis was performed in 2% agarose in a TBE buffer (45 mm Tris-borate, 1 mm EDTA, pH 8).

### 2.6 Statistics

The correlation analysis has been made by Pearson through the R-studio program.

## 3 Result

### 3.1 Greenhouse experiments

**Fig. 4:**
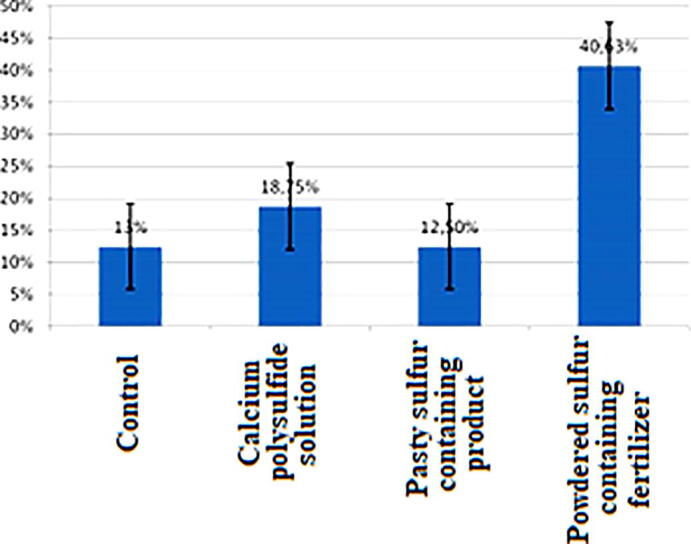
The effect of sulfur-containing products on the germination of wheat seeds, %.

The diagram shows that when growing wheat with various sulfur-containing drugs, the maximum germination rate was 40.63% when using powdered sulfur-containing organic preparations. The minimum percentage of seed germination was shown by the option with pasty sulfur-containing nano-particles of 12.50%. The solution of calcium polysulfide had a better effect compared to the control. The solution and powdered composition have a positive effect on germination, especially with powdered chemicals, the germination of wheat exceeds almost 3 times compared to the control (Fig. 4).

**Fig. 5:**
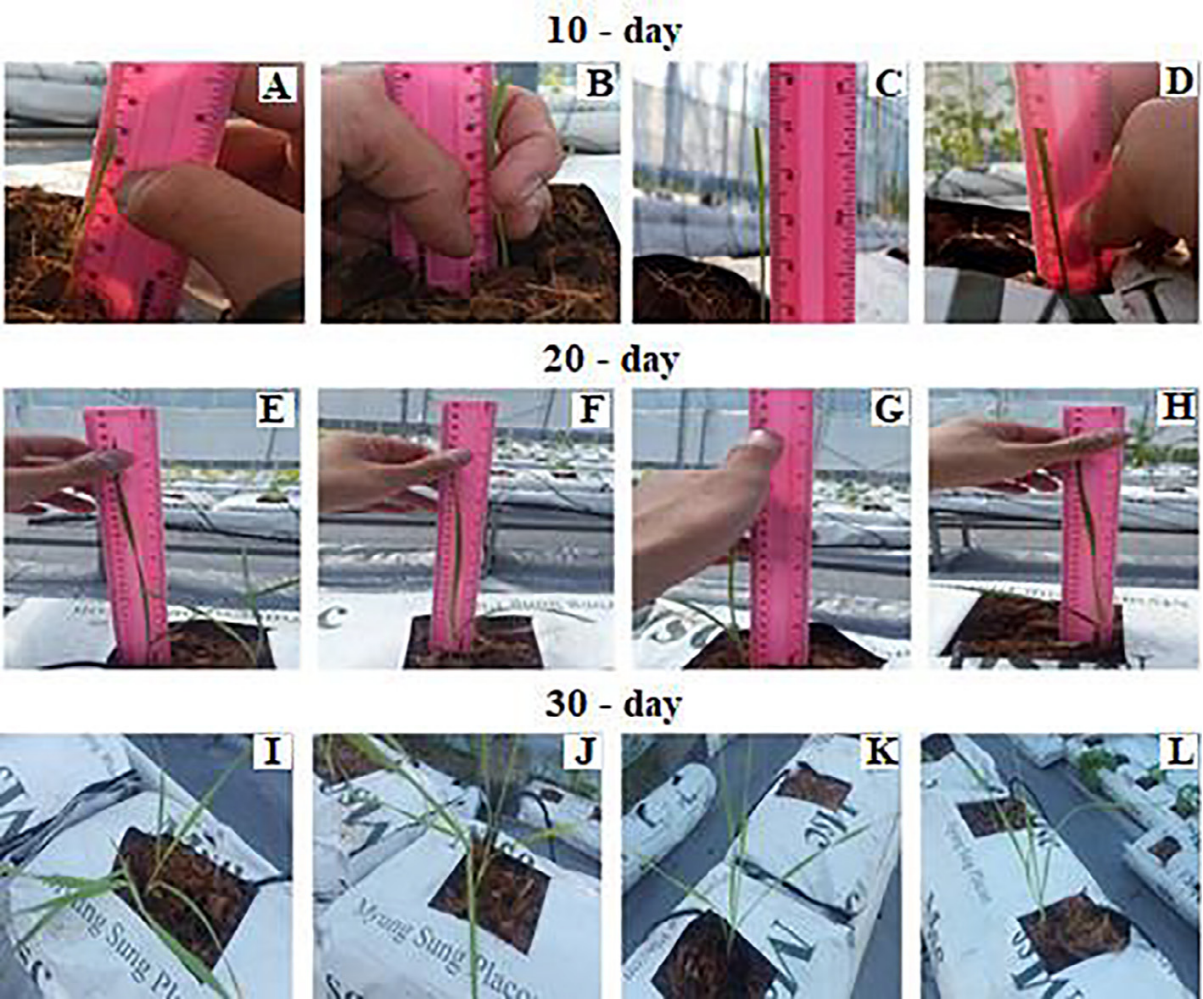
Control (A, E, I); Calcium polysulfide solution (B,F,J); Pasty sulfur-containing product (C,G,K); Powdered sulfur-containing organic fertilizer (D,H,L).

Seedlings appeared on the sixth day after planting, seedlings in the variants with sulfur-containing agrochemicals grew better than plants treated with water. Pasty compound at the beginning had a favorable effect on the growth and development of seedlings, but after a while began to lag behind other options. The highest data were expressed in the solution and powdered drug variants (Fig. 5).

Phenological observation showed that the sulfur-containing nano-particle has a favorable effect in the tillering, tubing, earing, and flowering phases until the grains are fully matured.

The area of the flag leaf determines the length of the spike and the number of spikelets. The average size of the flag sheet in length and width was superior in the variants with sulfur-containing preparations. If in the control version the sheet plate in length and width showed the lowest result of 9.92 ± 0.5 cm × 0.67 ± 0.04 cm, the high indicator in the solution version of the flag sheet reached up to 13.75 ± 0.7 cm × 0.75 ± 0.04 cm. The length and width of the flag sheet in the version with powdered composition is 12.4 ± 0.6 cm × 0.74 ± 0.08 cm. In the version with pasty gray, the length and width of the flag sheet were 10.7 ± 0.4 cm × 0.7 ± 0.06 cm.

**Fig. 6:**
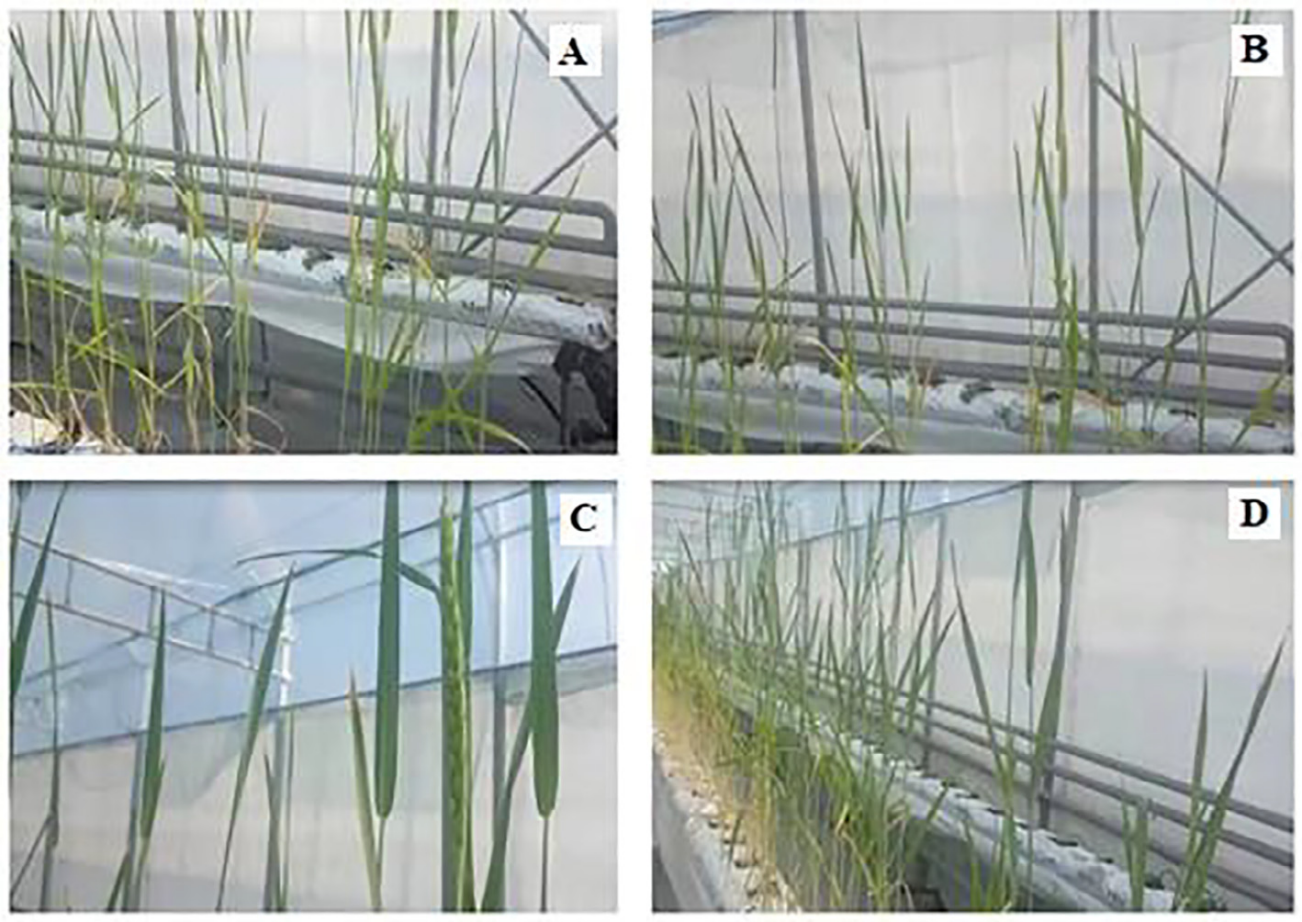
Formation of the wheat growth phase: control (A); Solution of calcium polysulfide (C); Pasty sulfur-containing product (B); Powdered sulfur-containing organic compound (D).

During studying in three re-growing wheat with different chemicals, it was found that the period of full maturity of wheat was 3 weeks earlier in the variants using dry and a solution of sulfur-containing fertilizer. Sulfur-containing agrochemicals, except pasty nano-sulfur, increase the productivity of wheat (Fig. 6).

**Fig. 7:**
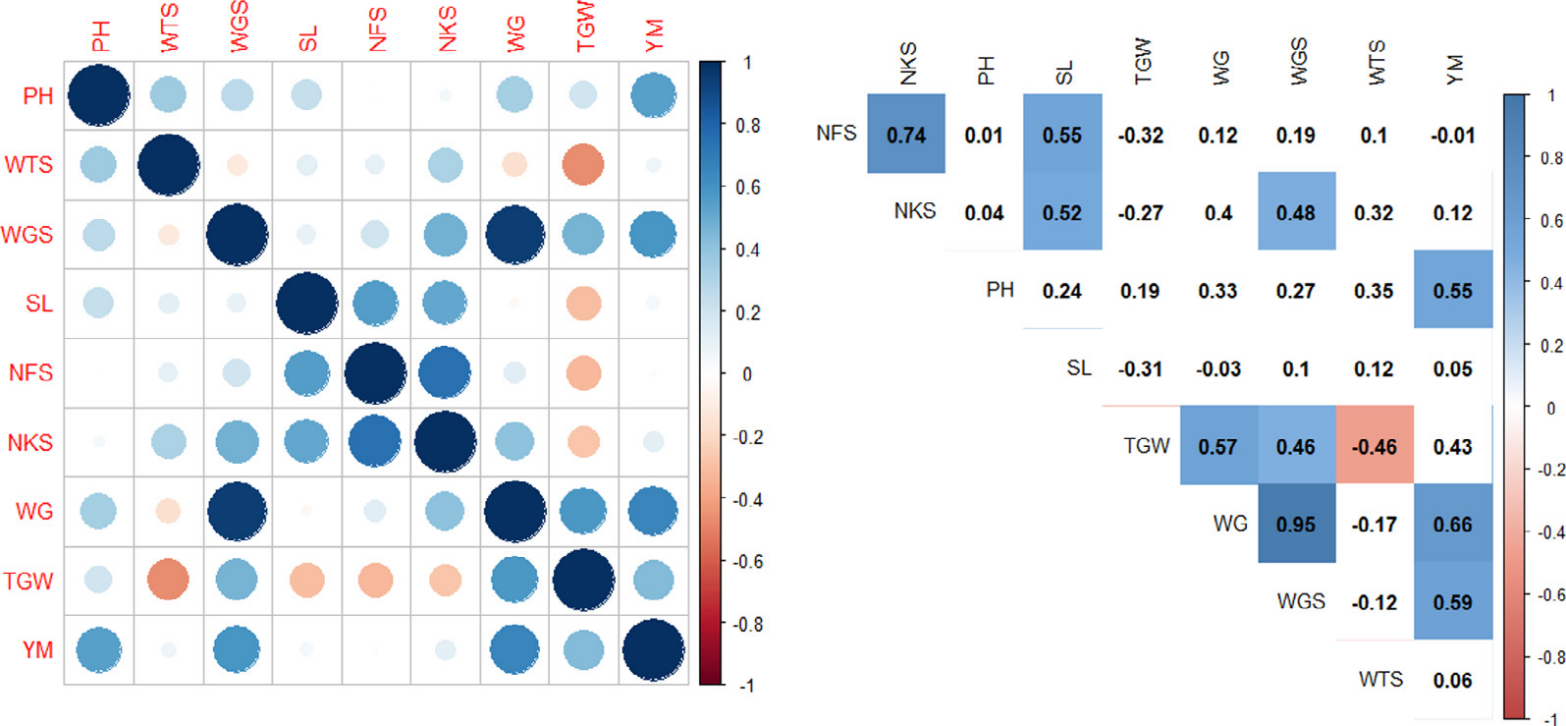
Correlation analysis of traits experiments in the greenhouse. Note: Correlations with P < 0.05 are highlighted in color. The color indicates either positive (blue) or negative (red) correlation. (Plant height, cm – PH, the weight of 100 spikes, gram – WTS, the weight of 1-grain spike, gram – WGS, spike length, cm – SL, Number of spikelets – NFS, Number of grains – NKS, the weight of grains, gram – WG, 1000 grain weight, gram – TGW, grain yield in grams per plot – YM.

As a result of structural analysis of all varieties taken for the study, statistical processing was performed. The indicators of structural analysis versions had a higher result in the sulfur-containing compared with control version. In addition, resistance to rust fungi was determined in versions with the addition of sulfur (Fig. 7).

During the study, it was found that the introduction of sulfur-containing chemicals increased the number of productive shoots, the number and weight of grains compared to the control. The use of the new drug as a preparation provided early maturation of wheat for 3 weeks.

Wheat does not have time to ripen where winter comes early and the additional amount of sulfur contributes to its rapid growth, development, and increase in harvest in the Northern regions of Kazakhstan. The results of the study show that the new sulfur-containing nano-particles are effective and profitable for obtaining an early and high, sustainable crop and can be used in agriculture.

### 3.2 Experiment in the nursery

Wheat varieties were grown using three versions of the new sulfur-containing composition in the nursery. The growth and development of wheat varieties have been studied in various variants. Plants in the control group were treated with water. A careful structural analysis is made in full ripeness that includes plant height, length of the spike, weighed the weight of 100 spikes, the weight of one spike, the weight of grain, weight of 1000 grains, counted the number of spikelets, number of grains in one spike. The grain yield is determined as well. Brown rust lesions were taken into account. The harvest of wheat significantly increased under the effect of using sulfur-containing chemicals compared to the control. It was found that the incidence of brown rust was significantly lower in the variants with the use of nano-sulfur.

Table 1 shows the average yield for three years of wheat varieties grown in different conditions in the nursery. It was noted that the damage to the rust fungus was high in the control variant, and it was also found that the yield was reduced by the rust fungus. During the analysis of all varieties, it was found that in the variants with the addition of sulfur, the damage to the rust fungus is small and the harvest is high. During these 3 years, it was experimentally proved that in all the studied varieties in the version with the addition of sulfur, the yield is high and the resistance to rust increases.

**Table 1:** Effect of sulfur-containing fertilizer on wheat yield, g/plot.

Varieties	Powder sulfur-containing agrochemicals	Sulfur-containing solute compounds	Pasty sulfur-containing drugs	Average value with sulfur-containing nano-particle	Control
Samgau	269,65 ± 9.48	248,94 ± 6.14	155,69 ± 3.87	224,76 ± 4.37	171,14 ± 1.79
Zhenis	327,80 ± 9.91	305,47 ± 9.64	250,38 ± 6.53	294,55 ± 8.64	127,30 ± 1.69
Raminal	255,62 ± 9.64	267,95 ± 8.07	129,82 ± 2.96	256,17 ± 7.39	138,16 ± 1.77
Kazakhstanskaya 9	269,54 ± 8.28	277,78 ± 6.51	224,87 ± 5.15	237,60 ± 5.52	264,18 ± 2.68
Kazakhstanskaya 16	238,50 ± 6.79	300,89 ± 9.12	210,22 ± 6.77	253,63 ± 6.14	315,26 ± 8.27
Lutescens 32	292,48 ± 7.84	287,51 ± 8.04	118,54 ± 3.18	241,36 ± 4.78	125,43 ± 1.79
Kazakhstanskaya 21	337,68 ± 9.93	332,31 ± 10.12	298,61 ± 6.69	277,86 ± 7.97	340,96 ± 9.12
Omskaya 29	259,18 ± 6.69	298,35 ± 9.45	217,48 ± 7.27	290,60 ± 8.90	141,90 ± 2.13
Koksu	299,51 ± 8.88	293,00 ± 9.71	202,78 ± 5.49	261,72 ± 5.98	219,80 ± 3.33
Zhadyra	237,34 ± 4.98	229,37 ± 6.52	112,67 ± 4.12	229,11 ± 4.61	129,27 ± 1.69
Morocco	239,87 ± 7.29	235,44 ± 5.45	108,44 ± 3.57	193,86 ± 5.18	114,92 ± 1.78
RL6040 (Lr19)	205,11 ± 5.97	201,74 ± 4.77	160,58 ± 5.62	191,86 ± 4.91	150,71 ± 2.42
RL6079 (Lr28)	273,20 ± 6.54	259,63 ± 6.58	191,34 ± 6.15	215,27 ± 5.12	200,49 ± 3.96
RL6082 (Lr35)	263,00 ± 7.83	256,38 ± 5.56	164,21 ± 4.28	234,63 ± 5.67	173,82 ± 2.94
Parula (*Lr68*)	252,98 ± 6.12	261,38 ± 6.48	152,79 ± 3.79	225,12 ± 4.84	174,05 ± 3.01
Lr39	301,43 ± 9.48	298,60 ± 9.79	155,42 ± 4.02	237,10 ± 4.03	176,17 ± 3.08
RL6081(Lr37)	186,37 ± 4.52	178,32 ± 4.14	138,94 ± 3.92	209,85 ± 2.98	150,41 ± 2.12

According to the structural analysis of wheat varieties grown in the nursery, it was found that when studying the harvest of wheat grain weight in one plot, the average yield of sulfur-containing variants is higher than that of the control variant. It should be noted that the decrease in yield in the control version indicates the effect of brown rust on the yield of the studied samples. Also, the favorable effect of the sulfur-containing preparation contributes to an increase in harvest and to a decrease in wheat disease.

**Fig. 8:**
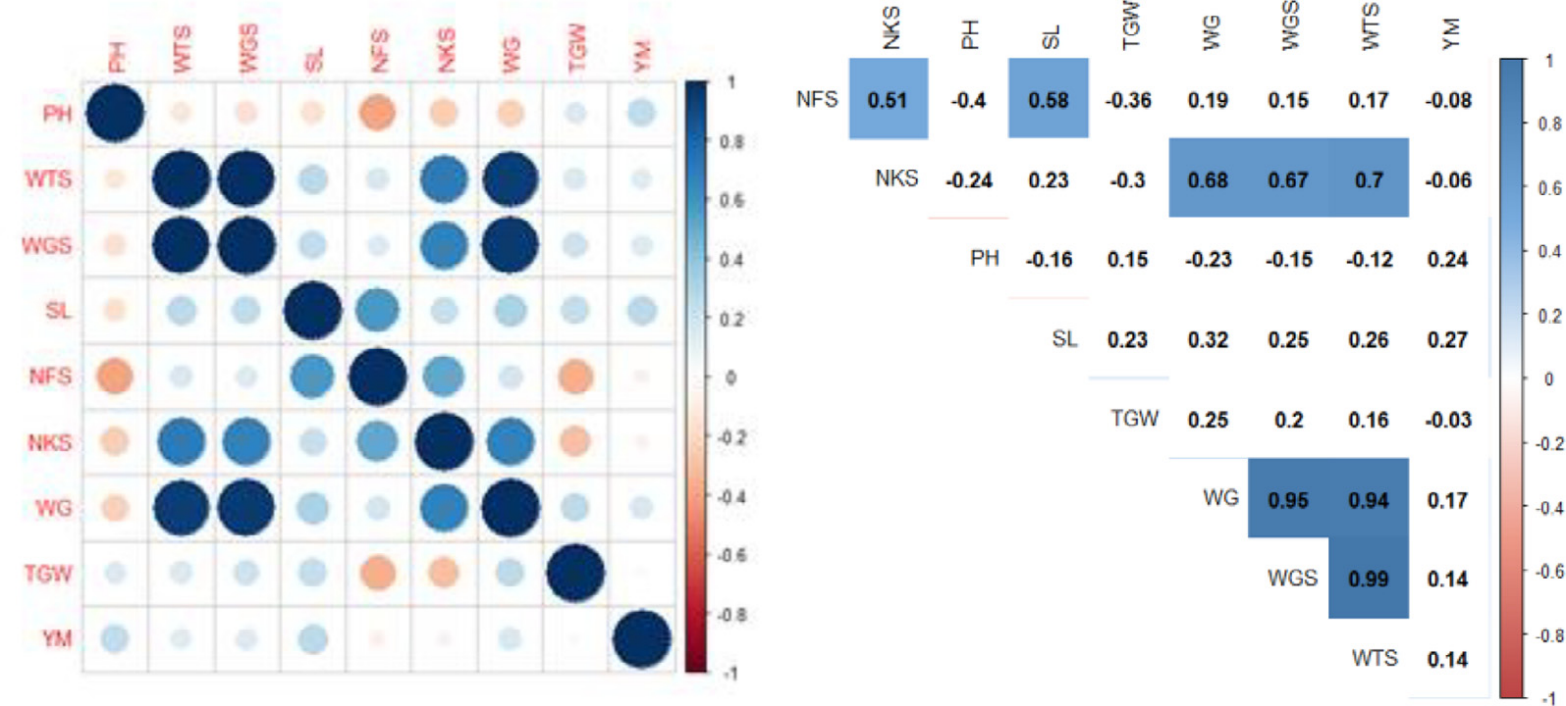
Correlation analysis of traits experiments in nursery-garden. Note: Correlations with P < 0.05 are highlighted in color. The color indicates either positive (blue) or negative (red) correlation. (Plant height, cm – PH, the weight of 100 spikes, gram – WTS, the weight of 1-grain spike, gram – WGS, spike length, cm – SL, Number of spikelets – NFS, Number of grains – NKS, the weight of grains, gram- WG, 1000 grain weight, gram – TGW, grain yield in grams per plot – YM.

The statistical processing was carried out as a result of structural analysis of all varieties taken for the study grown in the nursery (Fig. 8).

In the course of the study, compositions with the addition of sulfur in dry and dissolved form showed a high yield, compared to pasty sulfur and control. Therefore, chemicals with the addition of nanostructured sulfur in the dry and dissolved form are recommended to increase the harvest and stability of wheat varieties.

### 3.3 Molecular genetic analysis of wheat resistance to brown rust

Based on the analysis of the international databases GrainGenes, MASWheat, KOMUGI, the selection of molecular markers (6 PCs.) linked to the genes of resistance to brown rust was made (Table 2).

**Table 2:** Molecular markers linked to brown rust resistance genes.

*Lr-*genes	The name of the marker	The name of the marker	Nucleotide sequences of primers (5′-3′)	The expected size of the amplification product, bp	Literary source
Lr68	csGS	7B	5′- AAG ATT GTT CAC AGA TCC ATG TCA -3′ 5′- GAG TAT TCC GGC TCA AAA AGG -3′	385 bp	Herrera-Foessel et al. (2012)
Lr19/Sr25	PSY-E1	7D	5′- CTA CGT TGC GGG CAC CGT T 3′ 5′- AGA GAA AAC CAT TGC ATC TGT A -3′	191 bp	Yu, Sorrels & Dubcovsky https://maswheat.ucdavis.edu/
Lr35/Sr39	Sr39#50	2B	5′- CCA ATG AGG AGA TCA AAA CAA CC -3′ 5′- CTA GCA AGG ACC AAG CAA TCT TG -3′	250 bp	Mago et al. (2009)
Lr39	Xgwm 210	2D	5′TGCATCAAGAATAGTGTGGAAG 3′ 5′ TGAGAGGAAGGCTCACACCT 3′	182 bp	https://maswheat.ucdavis.edu
Lr37/Yr17/Sr38	Ventriup/ LN	2A	5′- GGT CGC CCT GGC TTG CAC CT-3′ 5′- TGC AGC TAC AGC AGT ATG TAC ACA AAA-3′	262 bp	Helguera et al. (2003)
Lr28	Wmc 313	4A	5′GCAGTCTAATCTGCTGGCG3′ 5′GAGGCTTGCATGTGCTTGA3′	320 bp	Bipinraj et al. (2011)

Based on the analysis of literature data, the selection of molecular markers (6 PCs.) linked to the genes of resistance to brown rust was made. PCR analysis protocols for identifying carriers of the brown rust resistance genes Lr68, Lr19/Sr25, Lr35/Sr39, Lr37/Yr17/Sr38, Lr39, and Lr28 were developed (Table 3).

**Table 3:** Protocols for PCR using primers for the corresponding LR resistance genes.

Primer	Gene	Initial denaturation (°C, min/s)	Number of cycles	Denaturation (°C, s)	Annealing (°C, s)	Extent (°C, s)	Last extent (°C, min)
csGS	Lr68	93(1)	30	93(30)	60(60)	72(60)	72(5)
PSY-E1	Lr19/Sr25	94(4)	10	94(20)	63(30)	72(1,20)	72(7)
			35	94(20)	58(20)	72(1,20)	
Sr39#50	Lr35/Sr39	95(10)	7	94(30)	60(30)	72(40)	20(60)
			35	94(30)	58(30)	72(40)	
Xgwm 210	Lr39	94(3)	35	94(1)	60(60)	72(60)	72(5)
Ventriup/ LN	Lr37/Yr17/Sr38	94(10)	30	94(45)	65(30)	72(60)	72(7)
Wmc 313	Lr28	94(3)	35	94(30)	51(30)	72(60)	72(5)

**Fig. 9:**
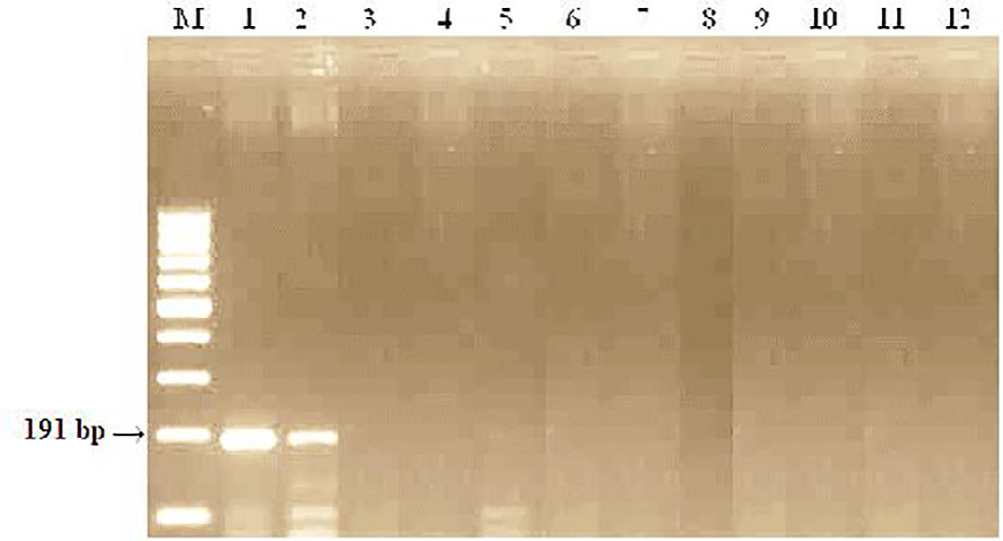
DNA amplification Products of samples from the control nursery (CP) of spring wheat using the PSY-E1 marker linked to the Lr19/Sr25 gene. M−Molecular weight marker (Gene-Ruler 100 bp DNA Ladder), 1- RL6079 (Lr28), 2- Zhenis, 3- Kazakhstan 9, 4- Morocco, 5- Raminal, 6- Lutescens 32, 7-Kazakhstan 21, 8 -Omskaya 29, 9- Samgau, 10-Zhadyra, 11 - Kazakhstanskaya 16, 12- Koksu.

PCR analyses were performed to identify carriers of brown rust resistance genes. Polymerase chain reaction (PCR) was used to identify carriers of resistance genes. PCR amplification was performed using PSY-E1 primers, the expected size of the amplification fragment is 191 bp (Fig. 9).

When PSY-E1 primers were used for PCR from 12 samples in the Omskaya 29 variety and the Lr19 control, the presence of an amplification product with a size of about 191 bp. was noted. Amplification products were absent in 10 samples.

**Fig. 10:**
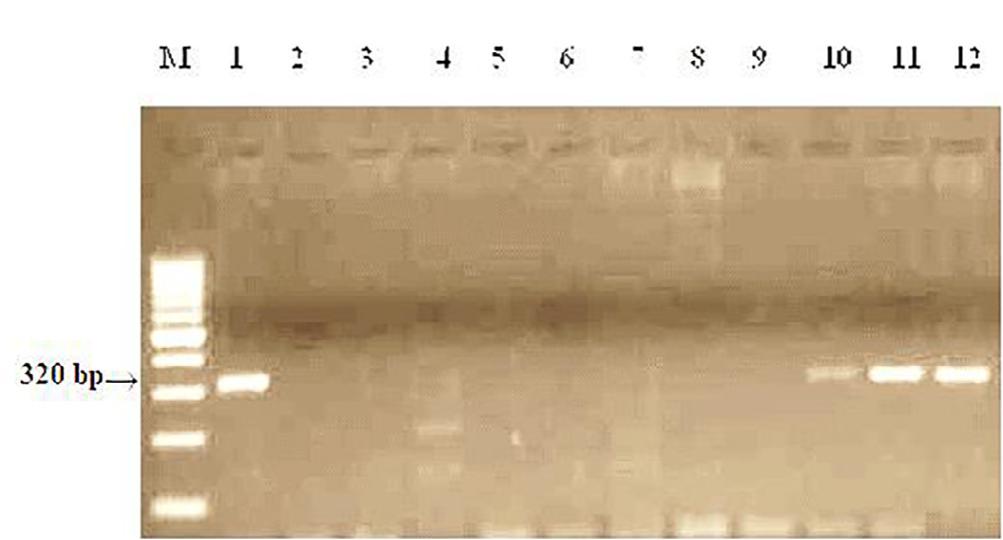
Products of amplification of DNA samples using primers to the wmc313 locus linked to the Lr28gene. M−Molecular weight marker (Gene-Ruler 100 bp DNA Ladder), 1- RL6079 (Lr28), 2- Zhenis, 3- Kazakhstan 9, 4- Morocco, 5- Raminal, 6- Lutescens 32, 7-Kazakhstan 21, 8 -Omskaya 29, 9- Samgau, 10-Zhadyra, 11 - Kazakhstanskaya 16, 12- Koksu.

The wmc313 marker was used to identify the resistance gene Lr28 from 12 common wheat samples. The expected fragment of amplification is 320 bp (Fig. 10).

Three samples (Zhadyra, Kazakhstanskaya 16, Koksu) and control Lr28 RL6079 showed the presence of an amplification product with a size of about 320 bp in the result of using primers wmc313 for PCR from 12 samples. These samples are carriers of the Lr28 resistance gene. Amplification products were absent in 8 samples.

The Lr35 gene is linked to the stem rust resistance gene Sr39 and is localized on chromosome 2B, the gene is sourced from *Aegilops speltoides* L., the test line is cultivar Thatcher RL5711. Lr35/Sr39 resistance genes to brown and stem rust are transformed from *Aegilops speltoides* L. PCR amplification was performed to identify carriers of the Lr35 gene using the STS Sr39 # 50 primers. Isogenic lines Lr35 RL6082 were used as a positive control for the identification of carriers of Lr genes.

**Fig. 11:**
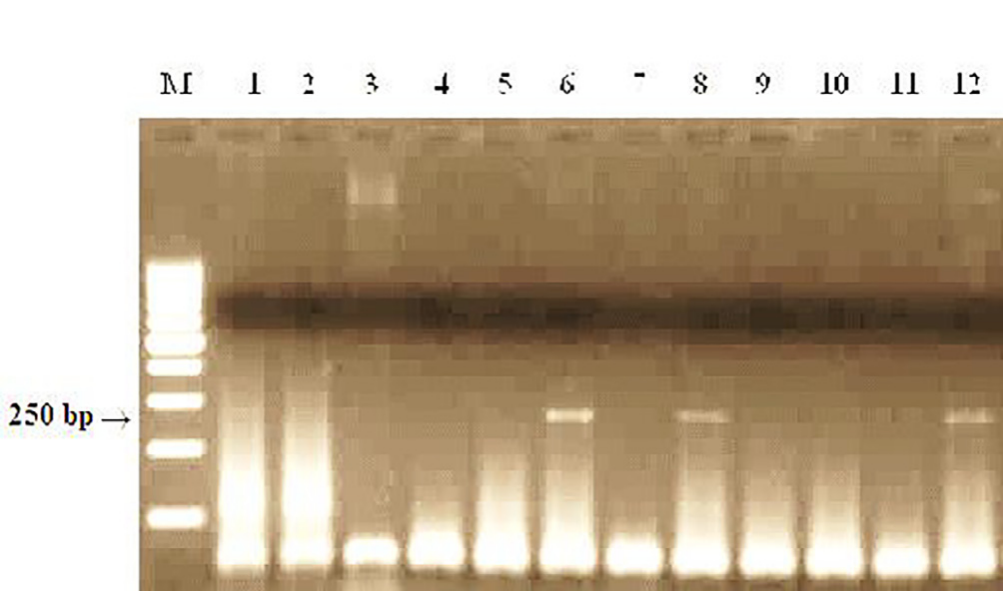
Products of DNA amplification of spring wheat samples using primers to the Sr39#50, locus linked to the Lr35/Sr39 gene. M−Marker of molecular weight (Gene-Ruler 100 bp DNA Ladder), 1-Morocco, 2- Zhenis, 3- Kazakhstanskaya 9, 4- Samgau, 5- Raminal, 6- RL6082 (Lr35), 7-Kazakhstanskaya 21, 8- Omskaya 29, 9- Koksu, 10-Zhadyra, 11 - Kazakhstanskaya 16, 12- Lutescens 32.

As a result of PCR analysis, it was shown that a DNA fragment characteristic of carriers of the Lr35 gene with a size of 250 bp formed in sample 2 and the isogenic line RL6082 (Fig. 11). The products of the characteristic amplification fragment were absent in 10 wheat lines.

**Fig. 12:**
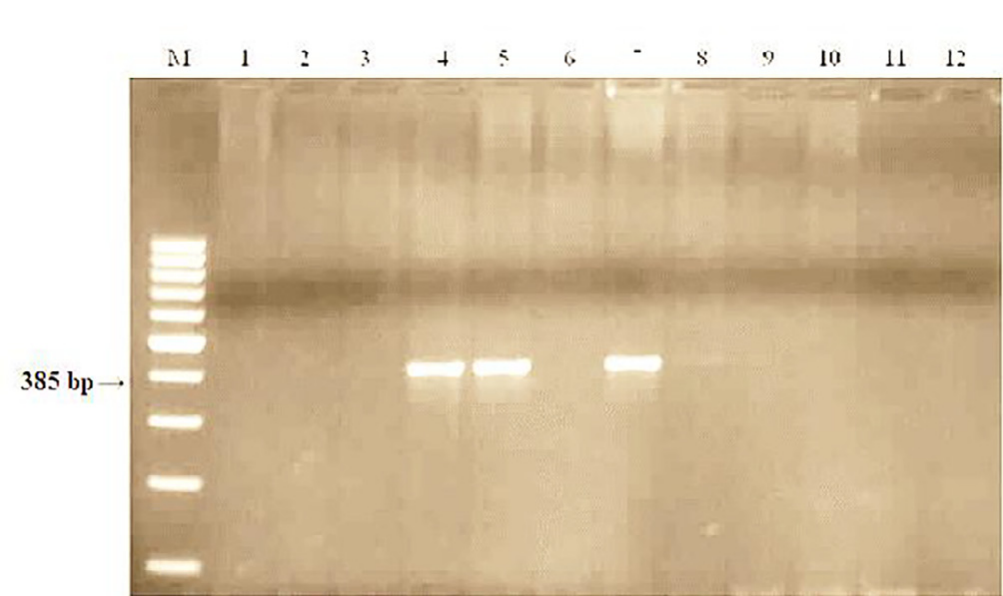
Products of DNA amplification of winter wheat samples using primers to the csGS locus linked to the Lr68 gene. M−Marker of molecular weight (Gene-Ruler 100 bp DNA Ladder), 1- Samgau, 2- Zhenis, 3- Kazakhstanskaya 9, 4- Parula (Lr68), 5- Raminal, 6- Lutescens 32, 7-Kazakhstanskaya 21, 8 -Omskaya 29, 9- Koksu, 10-Zhadyra, 11 - Kazakhstanskaya 16, 12- Morocco.

STS primers of the csGS marker were used to search for the *Lr68* gene. Fig. 12 shows the results of electrophoresis of PCR products, reflecting the presence or absence of the gene *Lr68* gene in the studied samples.

PCR analysis showed that a 385p DNA fragment characteristic of carriers of the Lr68 gene. n. formed in the Omskaya 29, Raminal, and Parula varieties. The products of the characteristic amplification fragment were absent in 9 lines.

**Fig. 13:**
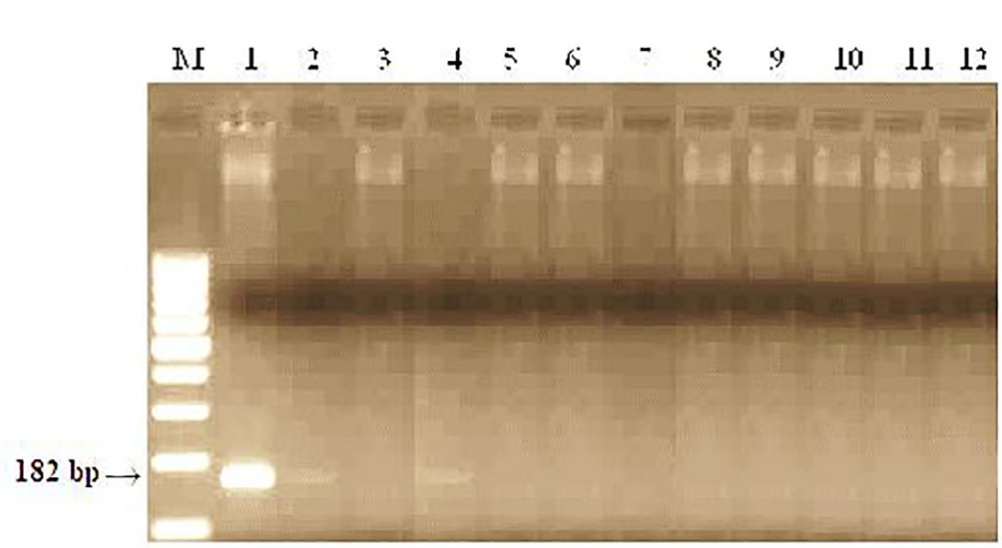
PCR analysis for identification Lr39 resistance gene using Venrriup/LN and Xgwm 210 markers. *1- Lr39 (RL6040),* 2- Zhenis, 3- Kazakhstanskaya 9, 4- Samgau, 5- Raminal, 6- Lutescens 32, 7-Kazakhstanskaya 21, 8-Omskaya 29, 9- Koksu, 10-Zhadyra, 11 - Kazakhstanskaya 16, 12- Morocco.

We also carried out PCR analysis to identify the Lr39 resistance gene and Lr37/Yr17/Sr38 resistance complex genes to leaf rust using the Ventriup/LN and Xgwm 210 markers (Fig. 13). As a result, of the 12 wheat lines studied, the Lr39 resistance gene was found only in the control, and the complex resistance genes Lr37 / Yr17 / Sr38 were found in one wheat cultivar.

The results of the phytopathological assessment of resistance to leaf rust in 10 samples of winter and spring wheat were carried out at the stages of seedling and adult plants. Genotypes are grouped into 4 groups: immune, resistant, medium-resistant, and medium-sensitive (Table 4). The group of immune cultivars (R) included 3 accessions that did not show symptoms of *Puccinia recondita* f. sp. Tritici, these include Raminal, Kazakhstanskaya 16, and Zhadyra.

**Table 4:** Phytopathological assessment of the resistance and molecular screening of promising lines to wheat leaf rust.

Varieties	Resistance to brown rust in natural conditions	Resistance to brown rust in artificial infectious medium		*Lr28* 320 bp	*Lr68* 385 bp	*Lr19/Sr25* 191 bp	*Lr35/Sr39* 250 bp	*Lr39* 182 bp	*Lr37/Yr17/Sr38* 262 bp
		Adult resistance	Germination resistance						
Samgau	60MS	30S	3	–	–	–	–	–	–
Zhenis	30MS	90S	4	–	–	–	–	–	–
Raminal	0	20MS	2	–	+	–	–	–	–
Kazakhstanskaya 9	30MR	20MS	3	–	–	–	–	–	–
Kazakhstanskaya 16	0	20MS	3	+	–	–	–	–	–
Lutescens 32	10MR	10MS	4	–	–	–	+	–	–
Kazakhstanskaya 21	5MR	5MS	4	–	–	–	–	–	–
Omskaya 29	10MR	10MS	3	–	+	+	+	–	+
Koksu	10MR	0	1	+	–	–	–	–	–
Zhadyra	0	0	x	+	–	–	–	–	–
Morocco	90S	100S	4	–	–	–	–	–	–
RL6040 (Lr19)	0	0	;	–	–	+	–	–	–
RL6079 (Lr28)	0	0	0	+	–	–	–	–	–
RL6082 (Lr35)	0	5MR	3	–	–	–	+	–	–
Parula (*Lr68*)	0	0	0	–	+	–	–	–	–
Lr39	0	0	0	–	–	–	–	+	–
RL6081(Lr37)	0	0	0	–	–	–	–	–	+

Table 4 shows the results of phytopathological assessment for brown and yellow wheat rust at the seedling stage in the greenhouse. 12 days after inoculation, the first symptoms of the disease appeared on the susceptible standard Morocco-55. Based on the table data, it can be concluded that out of 10 samples of spring and winter wheat studied, only 1 Raminal variety, which was affected by 2 points, can be attributed to moderate resistance to brown rust at the seedling stage. Defeat in 3 points was noted in 4 varieties of Kazakhstan 9, Kazakhstan 16, Omskaya 29, and Samgau. The highest number (3) was observed in the susceptible group of varieties: Kazakhstanskaya 21, Lutescens 32, and Zhenis. The rating of these varieties to brown rust was at the level of 4 points. Small brown rust pustules surrounded by necrosis were observed on the leaves of the isogenic line RL6082 (Lr35), so they are considered resistant (MR). Samples Raminal, Kazakhstanskaya 9, Kazakhstanskaya 16, Lutescens 32, Kazakhstanskaya 21, and Omsk 29 have a medium-susceptible reaction characterized by an infectious type (MS). Of the 10 samples of spring and winter wheat studied, the Samgau and Zhenis samples showed a susceptible reaction with an infectious type (S).

As a result of molecular screening for the assessment of resistance to the brown rust pathogen, 10 samples were presented. Using the wmc313 marker, the Lr28 resistance gene was detected in 3 samples (Kazakhstan 16, Koksu, and Zhadyra) out of 50 samples studied. Using the marker, csGS R/F from the studied samples, 2 identified the lr68 resistance gene. Molecular analysis using the PSY-E1 marker revealed the lr19 resistance gene in one of the 50 samples. Out of 10 samples using the Sr39#50 marker, 2 samples showed the lr35 resistance gene. The clamped Lr37/Yr17 gene was detected in the Omskaya 29 variety. Using the xgwm210 marker, No Lr39 resistance gene was detected out of 10 samples. The Omskaya 29 variety has 4 resistance genes in its genotype – Lr19, Lr35, Lr37, and Lr68. Thus, these lines are the most promising in terms of resistance to wheat brown rust

Thus, as a result of phytopathological assessment and molecular screening of the studied 10 samples of spring and winter wheat, 3 samples showed an immune response during phytopathological analysis, and molecular analysis revealed the resistance genes *Lr28* and *Lr68* in the genotypes of these lines. Out of 10 samples of spring wheat, Omsk 29 showed an immune response according to phytopathological assessment and 4 resistance genes were detected by molecular screening. Thus, in the future, this line can be used as a donor in the creation of new varieties resistant to diseases. The results obtained will be used in Kazakhstan to create brown rust-resistant wheat varieties using MAS-selection.

## 4 Discussion

It is determined that the application of nano-sulfur on soils with low sulfur content increases the coefficients of the use of nutrients from fertilizers, accelerates their outflow from the vegetative organs to the grain. The sulfur agrochemical affects the nitrogen metabolism in wheat plants, plays an essential role from the earliest stages of development in the metabolism in the plant cell which is closely interrelated with nitrogen cycle since both elements are mandatory components of proteins. If there is a lack of one of two elements, protein synthesis is delayed, it may stop altogether in the absence of both (nitrogen & sulfur) available sources for plants (Maslova, 1993).

An indicator of the sulfur status in plants significantly is correlated with bioavailable sulfur in the soil. Sulfur indicator in the biomass of shoots as follows: it affects the concentration of sulfur, the mass ratio of nitrogen to sulfur (N / S), the mass ratio of phosphorus to sulfur, and the sulfur nutrition index. Bioavailable sulfur in the soil significantly is correlated with the ratio of nitrogen to sulfur on the shoots of winter wheat and winter rapeseed (Sedlar et al., 2020).

Crops need nutrients for high harvests; however, they can only absorb ionic forms of the elements. At this stage, microorganisms are useful because they convert organically bound nitrogen, phosphorus, and sulfur into the soluble ions such as NH_4_+, NO_3_–, H_2_PO_4_+, HPO_4_– and SO_4_–. Mineralization is the transformation of organic compounds into inorganic compounds, which is a biological process that depends on temperature, precipitation, soil properties, the chemical composition of crop residues, the structure and composition of microbial communities, and the C:N ratio in the soil after the application of plant material. Adjusting the values of these factors allows to determine the rate and direction of mineralization of crop residues in the soil (Grzyb et al., 2020).

Recommendations are not well developed for testing the soil on sulfur content in the cultivation of crops in arid areas. In order to assess value of sulfur and nitrogen content in the soil and tissue for sulfur deficiency prediction on sites, it is obviously observed on morphology since the two minerals often are correlated. Therefore, there is a probable increase of a response to the application of sulfur. A supportive use of the N:S ratio is recommended, which may indicate an S deficiency for both barley and wheat (Conyers & Holland, 2020).

Sulfur dioxide (SO_2_) plays a useful role in protecting plants from environmental stress. Analysis of transcription factor gene expression showed that SO_2_ pretreatment reduced TaNAC69 expression, but TaERF1 and TaMYB30 expression changed slightly and remained at a higher level in wheat seedlings in response to drought stress. Together, this study showed that SO_2_ increases the drought resistance of wheat seedlings by transmitting H2S signals, and provided a new strategy for increasing plant resistance to drought stress (Li et al., 2021).

The key to ensuring high soil fertility and increasing crop yields is balanced mineral nutrition for all elements, taking into account their content, distribution, and transformation in the soil (Kulhanek et al., 2014). Sulfur stands next to elements such as nitrogen, phosphorus, and potassium - the second proteinogenic after nitrogen. The lack of sulfur, as well as nitrogen, reduces the synthesis of proteins, while the external manifestation of sulfur starvation of plants almost coincides with the signs of a lack of nitrogen nutrition. Its absolute necessity for the processes of respiration, photosynthesis, nitrogen, and carbohydrate metabolism is established (Järvan, Edesi & Adamson, 2011).

The sulfur nutrition of plants was satisfied without additional efforts before, but now and in the future, the resources of its entry into the soil are reduced, and the need for it in agriculture is growing due to the increased demand for high-quality agricultural products. The main reasons for the increase in sulfur deficiency are the lower content of sulfur dioxide in the atmosphere, the increased use of highly concentrated and ballast-free sulfur-free compounds, higher crop harvests, and increased sulfur removal (Matraszek et al., 2015). As a result, the use of grain-free drugs has become a necessary condition for obtaining high yields. An increase in soybean harvest from the introduction of sulfur was found against the background of the use of N, P, K, Ca, and NPK. Sulfur has a positive effect on the formation of tuberculosis bacteria and the chemical composition of soybean plants (Brodowska and Kaczor, 2009, Järvan et al., 2008, Edmeades, 2003).

There is few researches on the effect of sulfur on the uptake of phosphorus and potassium by plants than on nitrogen, and the findings are often contradictory (Shkel, 1979, Tisdale, 1974). The positive effect of sulfur on the assimilation of phosphorus and potassium by plants along with nitrogen was observed in calcareous sod-podzolic soil (Tserling, 1990, Shevyakova, 1983, Shkel, 1979). The researchers explain the improvement of plant nutrition with phosphorus and potassium under the influence of sulfur nanoparticles in these cases by the increased mobility of soil elements under the influence of sulfuric acid (Svetlov et al., 1987, Archer, 1974). Thus, our research shows that the study of the influence of sulfur-containing preparations relevant and promising, but in practice was conducted in insufficient volume.

The effectiveness of sulfur preparations and their combinations in growing wheat in the conditions of greenhouses and nurseries confirmed information literary sources, thereby it is recommended for use in agriculture.

Fungal pathogens that cause wheat brown rust can cause crop losses of up to 50–60%. One of the most effective methods to prevent these losses is to create resistant varieties with high yield potential. Therefore, the main control strategy-genetic resistance-was used to control wheat rust diseases, especially leaf rust. So far, the host's genetic resistance remains the most effective (El-Orabey et al., 2019). Genetic analysis and recently used molecular markers are commonly used to identify rust resistance genes in wheat varieties, especially leaf rust. The molecular markers were time-saving compared to the two conventional methods and the results were obtained quickly. Currently, individual the Lr gene can be detected by specific molecular markers. For example, the Xgwm259 marker is used for LR detection 46 (William et al., 2003), but we chose a similar Xgwm250 for LR detection 39. In accordance with the concept of partial resistance by reducing the rate of epidemic development in the field, despite the susceptible type of infection. These two wheat varieties; Giza 171 and Misr 3 in the first category, which showed moderate resistance (Broers, 1989). In our study, the Omskaya 29 variety showed moderate resistance, so we can say that the variety has partial resistance genes.

## 5 Conclusion

Nanostructured sulfur-containing growth stimulators affected the germination of wheat seeds in greenhouses and nurseries. Positive interactions were observed for powdered and dissolved forms. The germination rate using the paste was low in all experiments. When the seeds were treated with a sulfur-containing growth stimulator in powder and solution forms in greenhouses and nurseries, the new sulfur-containing chemicals were quickly absorbed into the seeds, which accelerated their germination. In the case of the pasty form, the absorption of the nano-sulfur into the seeds was much slower. The effectiveness of the new fertilizers has been revealed and the obtained results confirm that the application of the developed sulfur-containing product ensures early ripeness and yield of wheat in the conditions of the greenhouse and nursery.

As a result of phytopathological evaluation and molecular screening, the studied wheat samples showed an immune response in phytopathological analysis, and molecular analysis revealed the resistance genes Lr28 and Lr68 in the genotypes of these lines. The sample of wheat Omskaya 29 showed an immune response according to phytopathological assessment, and molecular screening identified four resistance genes. Thus, in the future, this line can be used as a donor in the creation of new varieties that are resistant to diseases. The results obtained will be used in Kazakhstan to create brown rust-resistant wheat varieties using MAS selection.

## Funding

Granting program for researching BR05234566 “Development and testing of technologies for producing new sulfur-containing nanocomposites and preparations”.

## Declaration of Competing Interest

The authors declare that they have no known competing financial interests or personal relationships that could have appeared to influence the work reported in this paper.
